# How Did the Village Community Perceive and Draw Strategies Against the COVID-19 Pandemic? A Qualitative Exploration

**DOI:** 10.7759/cureus.16331

**Published:** 2021-07-12

**Authors:** Ananta Bhattacharyya, Mahendra M Reddy, Ravishankar Suryanarayana, Surahalli J Naresh, Prasanna B T Kamath

**Affiliations:** 1 Epidemiology and Public Health, Sri Devaraj Urs Medical College (SDUMC) Sri Devaraj Urs Academy of Higher Education and Research (SDUAHER), Kolar, IND; 2 Media and Communication Centre, Sri Devaraj Urs Academy of Higher Education and Research (SDUAHER), Kolar, IND; 3 Preventive Medicine • Epidemiology, Sri Devaraj Urs Medical College (SDUMC) Sri Devaraj Urs Academy of Higher Education and Research (SDUAHER), Kolar, IND; 4 Epidemiology and Public Health • Biostatistics, Sri Devaraj Urs Medical College (SDUMC) Sri Devaraj Urs Academy of Higher Education and Research (SDUAHER), Kolar, IND

**Keywords:** corona virus, covid-19, in-depth interview, qualitative research, rural health

## Abstract

Objectives

There is a lack of evidence about the difficulties faced by the villagers and the mechanisms they adopt to cope with the ongoing coronavirus disease 19 (COVID-19) pandemic. In this study, we tried to explore the various stressors experienced by the villagers and the coping mechanisms. We also tried to document the future strategies that could be adopted to address the current pandemic situation.

Methods

An exploratory, descriptive qualitative study was conducted in five purposively selected villages in the Kolar district of South India. We conducted face-to-face in-depth interviews among nine key informants, including personnel across various health, education, and administrative domains. Two investigators carried out a manual descriptive content analysis to identify the codes and categories under three broad themes. A hybrid approach was used for coding the respondents’ views in the most appropriate words/phrases.

Results

A total of 146 codes were identified and grouped into 19 different categories under three broad domains viz. ‘stressors’, ‘coping strategies’ and ‘suggestions for future actions’ for the existing COVID-19 pandemic. The stressors mainly were due to household level problems like finance management and familial disruptions. Coping mechanisms adopted include social capital, government support, judicial resource management, child marriages and apathetic attitude. The suggestions for future actions included an emphasis on the involvement of gram panchayats, adoption of the ‘stay in village’ concept, better communication framework and financial pooling for future exigencies.

Conclusion

The stressors due to COVID among villagers were mostly related to household level issues. The mechanisms adopted to cope up with the stressors included both positive and negative mechanisms. The suggestions for future actions mainly emphasized the involvement of gram panchayats

## Introduction

The pandemic caused by coronavirus disease 19 (COVID-19), apart from posing a great danger to public health, may also expose the existing social inequity. The majority of the Epidemics and Pandemics evolve rapidly. Communities play a significant role in preventing and reducing the impact of an epidemic and developing resilience to mitigate its impact. The way a community responds to an outbreak depends mostly on the existing socio-cultural milieu [1,2].

Social stress caused by the fallouts of the COVID-19 pandemic has many nuances, including travel restrictions and disruption of cultural celebrations, limited healthcare facilities, social distancing with friends and family, closure of places of entertainment and leisure, indefinite closure of schools, disruption of routine immunization activities [3]. Migrant workers who travelled to distant places for work have lost their job and are back home after being stranded without money in a far off place pose further stress to the villages [4].

India is a country where about two-thirds of the population live in villages and rely on agriculture for their living. As the virus transmission in villages happened late, the government strategies adopted in urban areas were blindly translated across the villages without proper planning. Due to this diversity, villagers’ responses are expected to be quite different from urban [5].

To minimize the damaging effects of the pandemic, there is an urgent need to study various stressors being faced by the village community and their intrinsic coping mechanisms to tackle them. There is little documented evidence assessing the coping mechanisms adopted in few occupational groups in India [6,7]. An insight into this sphere has the potential to provide evidence for the development and design of community-based interventions and a designation of roles that could be played by the individuals, communities and governmental agencies while facing the onslaught of the ongoing pandemic.

With this background, the current study was planned to explore the various stressors caused by the COVID-19 pandemic as experienced by the villagers and the coping mechanisms. We also tried to document the future strategies to mitigate the effects of a pandemic as perceived by them.

## Materials and methods

Study Design and Setting

An exploratory, descriptive qualitative approach was adopted to achieve the objective. This study was conducted in five purposively selected villages out of 20 inhabited by locals with a predominantly agrarian economy catered to by the Rural Health Training Center of a medical college in the Kolar district during June 2020. Apart from gram panchayat (village level local self-government), government-run secondary level schools, industrial training institutes, primary health centres, sub-centres, Anganwadi, and designated staff are available in the study area. A few of the villages have strong non-governmental organization (NGO) support as well. 

Sampling and Participants

These five villages were chosen purposively to get varied pictures of the current COVID-19 pandemic. One village was selected which was facing a containment situation; one village was selected based on the presence of NGO in that village for the last decade, one village was selected based on its previous performance as a model village, one village where alcoholism was a known 'social evil', one for its closeness to the nearby town and one for its good existing health system.

To get detailed information about stressors and the coping strategies for the COVID-19 pandemic, we decided to approach the critical informants at the village level instead of individual villagers. A purposive criteria based sampling strategy was used to select the Key informants to conduct in-depth face-to-face interviews (IDI). We tried to include people across different sections who are in contact with villagers and are well abreast about the situation of the villages. These included personnel across various domains of grass-root healthcare workers (Accredited Social Health Activist (ASHA) and Anganwadi Worker (AWW)), school management team (secretary), member of NGO, village head (ex-public workers), and current gram panchayat member. It was ensured that at least one key informant from each of the five villages was interviewed by the time data saturation occurred. 

Study Procedure

We used an IDI technique to capture the key informant's experience regarding the existing COVID situation and how the villagers acted upon it. The interview guide, along with probes to be used, was prepared in English. The interview guide was not translated into Kannada as the interviewer was fluent in both Kannada and English. This interview guide was flexible and was changed as per the experience of previous interviews based on the constant comparative analysis performed by the researchers.

In-depth interviews (total of nine IDIs to achieve data saturation) was done by a trained and experienced qualitative researcher who is a male medical doctor having a master degree in Community Medicine conversant in both the local language (Kannada) and English, and working in a private setup with no assigned role in the government sector. This allowed for an unbiased view concerning the government performance in this current situation. The interviews were conducted in a language (English/Kannada), time and places of respondents' convenience (workplace). Privacy was ensured, and there were no other members other than the respondent and interviewer. We did not have any participants refusing to participate. The interview was audio-recorded and later transcribed in the English language for analysis. The informed written consent and also consent for audio recording were obtained separately before the commencement of interviews. At the end of each interview, 'member checking was done by narrating the summary to the participants, and clarifications were sought and thereby validating the data collected during the interviews.

All investigators practised precautionary measures, including wearing N-95 masks, face shields, gloves and maintaining social distance throughout the interview procedure. All the participants were also provided with N-95 masks. Informed written consent was obtained before the start of the interview. The Institute Ethics Committee approved the study protocol of Sri Devaraj Urs Medical College, Kolar, Karnataka, India (SDUMC/KLR/IEC/123/2020-21).

Qualitative Analysis

Descriptive content analysis was carried out manually by the principal and co-principal investigators to identify the codes and categories under three broad themes (which were decided a priori based on the aim of the study). A constant comparative analysis technique was adopted to identify answers to the broad themes. We used open coding approach to code the transcripts. We used a hybrid approach, i.e. mixed coding (comprising both inductive and deductive), for coding the respondents' views in most appropriate words/phrases. The transcribed texts were read line by line thoroughly, and codes were assigned to paragraphs or segments of texts relevant to the broad topic of interest. The codes conveying a particular meaning were grouped into a category. The disagreements in coding between the two researchers were resolved through discussions, and a third investigator's opinion was sought in case of any unresolved disagreements. The final results were reported using categories along with verbatim quotes. The findings are reported as per the 'Consolidated Criteria for Reporting Qualitative Research (COREQ) [8].

## Results

**Figure 1:**
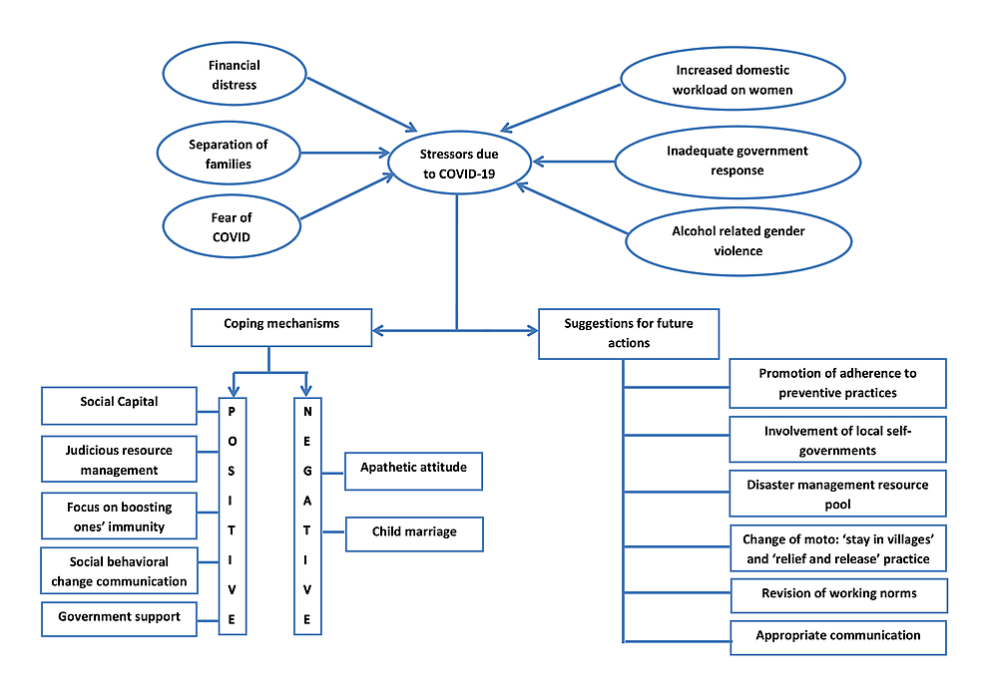
Schematic representation of qualitative findings – ‘themes’ and ‘categories’ as perceived by the key informants

We conducted nine in-depth interviews among the key informants from selected five villages. The duration of the interview ranged between 15 to 40 minutes. A total of 146 codes were identified and later grouped into 19 different categories under three broad domains viz. 'stressors', 'coping strategies' and 'suggestions for future actions' for the existing COVID-19 pandemic (see Figure 1).

Theme 1: ‘Stressors'

We found various stressors related to the COVID-19 pandemic among villagers under our study. These stressors were broadly divided into six different categories (see Table 1).

**Table 1:** Theme 1 – ‘Stressors’ due to COVID-19 among villagers in Kolar district, South India AWW - Anganwadi worker, COVID- corona virus disease, NGO- Non-governmental organization, Panchayat- Village level local self-government

Category	Verbatim quotes
I. Financial distress	“First 15-20 days they tried their level best and after that, they realized that lockdown and will not open up, they paid the labourers who came to work and finally the farmer was at a loss because he couldn’t sell the product.” (Panchayat member, 45-year-old, Male)
	“…whatever produce the farmers got because there was no price they buried their produce themselves, there was no rate for the produce… many actually came to the verge of committing suicide” (Ex-panchayat member, 60-year-old, male)
	“Industries are closed; a lot of youths who have done diplomas couldn’t do anything. They couldn’t do anything. Like I said whatever 100 rupees they used to spend in a day they had to use it for say five six-day now.” (Panchayat member, 45-year-old, Male)
	“…there are families who even cannot afford even one meal a day. There are about 40 families in my own village who have absolutely gone into abject poverty.” (AWW, 40 years)
	“…according to me, 75 to 80 families have gone into poverty…definitely no doubt… there is no earning nothing…” (Ex-panchayat member, 60-year-old, Male)
II. Separation of families	“Because of corona people who are in Bangalore and other cities have come back, the problems have started, as far as I know now two-three families have already divided their properties and all. Because of people coming back and staying together for two to three months it has come to that level that they had to separate themselves from their properties and all just because of this corona effect. That was really a surprise!!!” (Panchayat member, 45-year-old, Male)
III. Alcoholism linked gender violence	“…they (womenfolk) were quite happy, but once they opened (liquor shops) again they started, the wives tried there level best to stop saying that “from past one and half months you have not had stay like this it is quite good for us and our family” but the husbands didn’t like that… Physical abuse also started that time.” (Panchayat member, 45-year-old, Male)
IV. Increased domestic workload on women	“In households, the men do not go outside, they are at home, they want in time tiffin, lunch, snacks, coffee, tea, they demand and also children so definitely women feel burdened.” (Ex-panchayat member, 60-year-old, Male)
V. Inadequate Government Response	“For example, when a police are given an in-charge saying please don’t allow anyone to go, people used to call up and tell “leave him”, leave that vehicle, leave this vehicle. So, once that started, the police told ‘now ok when I am stopping some other fellow is coming and opening so let me also leave whomever I want, so they started leaving out people.” (Panchayat member, 45-year-old, Male)
	“…when they have gone there (quarantine facility) they didn’t have water to drink, no toilet facilities there, the hostel which was meant to stay. We called up the revenue department they were telling “yeah, we will get it cleaned”…If they don’t have water to drink, then how can they keep themselves clean? At least water to clean their hands…All that is dependent on the revenue department not on the health or police.” (Panchayat member, 45-year-old, Male)
	“…no revenue official has visited the village, no one ever came to speak or get to know the situation in last three months…” (NGO member, 65 years, Male)
VI. Fear of COVID	“…farmers fear to venture to markets to sell their produce now once the lockdown is clear…due to the possibility of getting COVID as people from all over assembles there…” (Panchayat member, 50 year, Male)
	“Doctors are there but they are afraid to go to them, see now, for example, someone has caught cold or headache then immediately he will be sent for quarantine or isolation… for this reason, many people prefer to be self-treated with home available traditional treatments.” (Ex-panchayat member, 55 years, Male)

Financial Distress

This was a major factor that caused stress among villagers. The loss was manifested among migrants returning to villages and locals into various professions like agriculture, sericulture, industry sector, and daily labourers, all affected due to loss of jobs. 

The key informants, in general, shared their concern that a sizeable number of families had plunged into poverty due to this COVID situation. There was displeasure due to the discriminatory practice of the distribution of Public Distribution System (PDS) even in this COVID situation, especially among the Above Poverty Line (APL) section that also lost jobs and financial insecurity them into poverty with even starvation. This situation even drove some to the extremes of ending their lives. 

Separation of Families

In few villages, there were instances wherein the families welcomed back their relatives who returned from their places of work. However, after an initial period of bonhomie, fissures appeared in their relationship, and few of them went to the extent of separating their properties and households.

Alcoholism Linked Gender Violence

There was a ban on alcohol during the lockdown period, and village women folk were happy about that. There were instances wherein alcoholics (in one village, rampant alcoholism in both genders) purchased the smuggled alcohol but later had to stay away from it because of the existing lockdown. Later, once there was a relaxation of lockdown, the women folk pestered their husbands not to revert to drinking but to stay away from it. This resulted in resentment and physical abuse of the womenfolk in villages.

Increased Domestic Workload on Women

As the gentlemen had to stay back at home without any work, they demanded to be served food and snacks even at odd times. The presence of additional members and children around the house deprived the women of rest and time for themselves.

Inadequate Government Response

There was a general resentment about the way the Government functionaries acted during this COVID situation. This grudge was more against the Revenue officials who acted in a highhanded manner. They failed to make proper arrangements for the distribution of provisions allocated by the government. Police, they felt, could not implement lockdown properly due to interference by the Revenue Officials. Though, by and large, the health officials were doing a satisfactory job, even some of them did not show enough sensitivity towards the community's concerns at times.

Fear of COVID

People in villages have developed a general sense of fear as they have come to know from various sources about the nature and dynamics of COVID and the non-availability of any definitive treatment modality. People are scared to visit any hospital for their usual health problems to fear being screened and then isolated.

Theme 2: 'Coping mechanisms'

To handle the current COVID crisis, the people in villages adopted various coping mechanisms to face this unprecedented onslaught in their way. These mechanisms were sometimes negative and, most of the time, positive. These various coping mechanisms were broadly divided into seven different categories (see Table 2).

**Table 2:** Theme 2 – ‘Coping mechanisms’ due to COVID-19 among villagers in Kolar district, South India AWW- Anganwadi worker, COVID- coronavirus disease, NGO- Non-governmental organization, TV- television, Panchayat- Village level local self-government

Category	Verbatim quotes
I. Social capital	“…we ourselves villagers get together, about 15 members collected money from people and headmen and shared and distributed for the needy whenever required, both money and also food grains…” (Panchayat member, 50-year-old, Male)
	“…many people have expressed they cannot withstand this economic stress anymore and take away their own lives…these times we have stood by them…spoken to people who were demanding money back from them…explaining the situation and also in some instances giving some money on their behest…” (Ex-panchayat member, 60-year-old, Male)
	“Some fruit vendors or vegetable vendors when they come, we ourselves stop them, check whether they have a mask, and other things and only then we allow them. If they do not have a mask we send them back…” (Panchayat member, 50-year-old, Male)
	“… a few years ago people had no food…one guy who had foodstuff at home…villagers decided to go to him and ask for food…he respondent immediately and gave food for the whole village for a month…that was a few years ago. But, because of value systems have changed…when someone came from outside, the whole villagers stood and objected and she was made to leave the village even though she was the daughter-in-law of the village headman…because of fear of COVID, she came from the red zone and thus was made to leave the same evening back…” (NGO member, 65 years, Male)
	“…In villages wherever NGOs work the social engineering is fantastic! Now villagers meet us every day which did not happen earlier…Since March 29^th^ we are meeting almost every day and discussing…that unity, social cohesion, social engineering in the village is taking very much because… Every crisis creates an opportunity… Thanks to COVID” (NGO member, 65 years, Male)
II. Apathetic attitude	“I think if the disease whatever that COVID-19 comes to the villages, if it starts affecting the people in the villages, I think that is the time these people will get frightened and start off whatever that social distancing, wearing of masks, cleaning of hands, I think that is the time they take importance, till that time I think these people are not taken it seriously till now… till today… whatever it is they have not taken it seriously.” (Panchayat member, 50-year-old, Male)
III. Judicious resource management	“…mostly they have taken loans from their owners or from their colleagues where they work…” (AWW, 45 years)
	“…Say they were spending on 100 rupees per day it came down to 15 – 20 rupees per day, they had to distribute that money over four five days that 100 rupees.” (Panchayat member, 45 year, Male)
IV. Focus on boosting one’s immunity	“…they (villagers) are making homemade decoctions and drinking, using ginger, pepper, tulsi (a type of medicinal herb)…these things they add and make preparations and drink…. As much as possible to drink hot water, have hot preparations…” (Ex-panchayat member, 60-year-old, Male)
V. Social Behavioral Change Communication	“Sir, in TV they used to show what will happen if we do not take these measures or follow these things, this caused fear among people and they themselves understood that and they themselves started buying masks and using them. In spite of us telling them, initially, it was not there they used to sit together and talk chewing their betel nut and leaves; this practice was more. After that, once they got to see in TV these awareness programs then they changed their behaviours.” (AWW, 45 years)
VI. Child marriage	“… a lot of child marriages are taking place…because everybody’s attention is something else…COVID 19. People take it as an excuse or opportunity whatever it is, to marry off their daughters….it is easy…not expensive…not much expense” (NGO member, 65 years, Male)
VII. Government support	“We are getting employment from gram panchayat. There is Mahatma Gandhi rural employment program, wherein people are working last few months without trouble. The only thing we need is that the bureaucracy delivers...I can assure whichever villages we (NGO) are working, I can assure there is no financial distress…” (NGO member, 65 years, Male)

Social Capital

Villagers are by and large a close-knit community with shared interests and practices. They are usually well known to each other. In situations of an emergency affecting the community, there is a sense of belonging and togetherness among them and a resolve to battle it together. In many scenarios, as quoted by the respondents, it was evident that the village men acted as the 'gate-keepers of the community. There were many examples wherein persons with better resources came to the rescue of the needy. The headmen of the village also stood by their community during this crisis.

NGO's who are part and parcel of the village system also provide an excellent social capital in the villages where they are present by facilitating the villagers to face the situation by creating a bridge between the government and the community. They have also trained the village folk, both men and women, in semi-skilled works that could be done at household, thereby increasing household financial security. Also, the NGO's have lent their hand in enabling the villagers to face the early crisis by supplementing the existing public distribution system.

Apathetic Attitude

We come across most of the study participants who expressed that some villagers were oblivious of the current problem and behaved nonchalantly, giving a negative influence among others who stood by the preventive guidelines. They outwardly showed no concern or fear towards COVID. This was more significant in the initial days and gradually reduced with more awareness over some time.

Judicious Resource Management

To get over the economic crisis raised due to the COVID situation, the villagers resorted to cut their non-essential expenditure and staggered the expenditure on essential requirements. The villagers initially relied on the savings, but as the same got depleted resorted to borrowings. Some of them who came back from different workplaces got along with the extra money by raising loans from their employers and colleagues to handle the uncertain developments. 

Focus on Boosting One's Immunity

Coming to terms that COVID does not have any definitive treatment or vaccine, the villagers tried to cope with this fact by resorting to practices that were perceived to boost immunity. Some of them took to physical exercise, while others sought homemade herbal preparations.

Social Behavioral Change Communication

Some of those had evinced callous attitudes towards COVID, gradually shown a change in their behaviour and attitude due to awareness messages received mainly via the mass media and to a certain extent via social media (village youth).

Child Marriage

The current situation has made the administration busy and focused on tackling the COVID crisis. Also, there is an existing ban on large social gatherings. As the economic crisis is looming large, some families took this opportunity to marry off their daughters below the marriageable age and thus save money.

Government Support

The state and the central government distributed rations that helped in tiding over an imminent food crisis. The timely implementation of the Mahatma Gandhi National Rural Employment Guarantee Act (MGNREGA) and local NGO support created local job opportunities that averted the villagers to go to places of risk of acquiring COVID also empowered them financially. As part of Public health services, ASHA workers paid visits to households to educate them on COVID and take stock of their health status.

Theme 3: 'Suggestions for future actions'

Based on the experience of tackling the COVID-19 situation in various capacities, our study participants came out with suggestions which, in their opinion, are to be acted on a priority basis (see Table 3).

**Table 3:** Theme 3 – ‘Suggestions for future actions’ due to COVID-19 among villagers in Kolar district, South India ASHA- Accredited Social Health Activist, COVID- coronavirus disease, MGNREGA- Mahatma Gandhi National Rural Employment Guarantee Act, NGO- Non-governmental organization, Panchayat- Village level local self-government

Category	Verbatim quotes
I. Promotion of adherence to preventive practices	“Although awareness is there from children to elderly…still there is a scope for improvement…Using ASHA or Anganwadi worker…making them visit house to house and reinforcing them is essential to keep these thing going (adherence to preventive practices)” ((Ex-panchayat member, 60-year-old, Male)
II. Involvement of local self-government	“Maybe lack of numbers, they don’t have so many people to do these works, may be given the responsibility to village panchayat members…and in the village, there will be one or two headmen. I think they should call them and tell them if anything happens please tell us and do it in few hours or say one day. They are not getting them involved; it’s like once they come ‘I am the boss’ you listen to me (government officials).” (Panchayat member, 45 years, Male)
	“I will go to local governments…instead of all approaching all villages one by one…approach the gram panchayats which have representatives from 15 to 20 villages. Make them understand the problem, equip them, build their capacity and then give them the challenge to face this COVID…Karnataka has 6000-gram panchayats…make use of them…” (NGO member, 65 years, Male)
III. Disaster management resource pool for future	“As an NGO we facilitate…they themselves for the first time in the village has formed a disaster management committee…they said – COVID is now, we don’t know what will happen after 10 years or 20 years. So, nobody in a village should suffer then with no food, no work, nothing…right? So why don’t we start a resource raising committee from now itself.” (NGO member, 65 years, Male)
IV. Change of moto: from ‘stay in households’ to ‘stay in villages’ and from ‘only relief’ to ‘relief and release’	“My suggestion would be…make them stay in villages rather than lock in houses, let them not go outside the village. When he or she is able to make 400 rupees per day here (means village), why should they go outside and get into this nonsense? (contract infection) With this MNREGA (they get 275 rupees per head per day) and NGO supporting it with 125 Rupees extra per head per day…they can easily make 400 rupees then why they have to go outside?” (NGO member, 65 years, Male)
	“We always believe in ‘relief and release’ approach, this is our main strategy…for example in this village we provided relief only for first one month…15 days once, twice in the first month after that we stopped relief…after that people started earning their own livelihoods and every week they are getting 2000 rupees each family with which they are buying their rations… it’s not going out (of the village)…the government program is their MGNREGA…using this they are working in natural resource management…I mean release people from the clutches of the situation…” (NGO member, 65 years, Male)
V. Revision of working norms	“There are poor people who do not have ration cards… the government must make things easier for them to get ration cards…even if some documents are found lacking…still they must be issued ration cards soon so that it would help them to at least get the supplies provided by the government” (School secretary, 40 years, Male)
VI. Appropriate communication	“If someone asks them something you must talk to them politely, its natural human psychology if someone talks rashly even they revert back in the same manner. So talk politely then everything will be solved.” (Panchayat member, 45-year-old, Male)

Suggestions

Promotion of Adherence to Preventive Practices

Although there is an existing level of awareness leading to practice among the village community, our respondents felt that there is still a scope to augment the level of awareness among them further. They suggested that healthcare workers like ASHA could visit households at least once a week to reinforce the preventive practices and be abreast with the evolving scenario. They also opined that officials from the revenue department should visit the community and gain a first-hand experience of their problems to chalk out a better remedial plan for the future.

Involvement of Local Self-Government

The respondents felt that local governments were being bypassed and their services not appropriately utilized. The local governments were not entrusted with any specific challenges or given guidelines to act upon. The local governments were mostly sidelined, and on certain occasions, they did not even know how to respond to this situation when approached by the villagers. Since the people representing these local self-governments are well aware of the local situations, they must be used to handle the problems faced by the villagers. There is a felt need for capacity building of the local self-governments to face these kinds of emergencies.

Disaster Management Resource Pool for Future

Prompted by the COVID crisis, one of the villages mooted an idea of raising funds for a possible similar future crisis. This innovative idea can be replicated in other places to become self-reliant and better prepared to face a disaster like the situation with full ownership of the people themselves.

Change of Moto: From ‘Stay in Households’ to ‘Stay in Villages’ and ‘Only Relief’ to ‘Relief and Release'

The NGO, with experience of working with local governments for more than 40 years, felt that some of their innovations could be a game-changer, such 'out of the box is a must to tackle such unprecedented events. He felt that the villagers need not be restricted in the households (the current COVID guidelines) but rather restrict themselves to their villages and be empowered to earn their livelihoods within their respective villages (thus reducing the inter-village or village to city movements) by making use of Government schemes like MGNREGA. He also emphasized the need to empower and use the local self-governments in tackling the existing COVID situations at the village level.

Revision of Working Norms

Most of the respondents suggested that the government guidelines for containment must be relaxed at least at the village level wherein instead of 'containment of villages' the norms must be changed to 'containment of households' as most of the activities like managing the livestock management, agricultural activities are external and come to a standstill, thereby, causing financial hardships if the current guidelines are in place.

Respondents expressed that the villagers are dissatisfied because of the differential norms in public distribution of food grains (APL people felt left out). The respondents also expressed to go back to the practice of the distribution of PDS as was done before the introduction of biometry in order to prevent the delay of distribution (happening due to deficient internet services in villages) and also to prevent over-crowding at the PDS shops, which happens due to increased waiting time in order to get their biometric approval. They also opined that due to the COVID situation, some households interested in PDS as hither-to-fore are now in need of it and must be facilitated by relaxing the norms to get the ration cards issued to them.

The respondents mentioned that the moratorium provided by the government in the form of deferment of payment of loans by three months must be considered for further extension without the imposition of any additional interest. 

Appropriate Communication

The key informants of the villagers felt that there was a clear lack of communication between the state government officials and the local panchayat members. The way the government officials behaved with the villagers during this crisis led to a clear dissatisfaction among them. This must be addressed on priority so that the government can gain back the confidence of the villagers and make them a partner to tackle this situation rather than turning them into a source of hindrance.

## Discussion

This study used key informants to understand the impact of the ongoing COVID-19 pandemic among villagers, particularly the stressors precipitated by it and their response to tackling these stressors. We also went a step ahead and found suggestions for the future that could be adopted to manage the situation better. The stressors mainly were related to household level problems like finance management, familial disruptions, gender violence and increased domestic workload on women folk. The villagers adopted few positive coping mechanisms like capitalizing on social bonds, government support, and judicial resource management. The negative mechanisms that were resorted to included child marriages and an apathetic attitude towards the situation, especially at the beginning of this pandemic. The suggestions for future actions included emphasizing the involvement of gram panchayats, adopting the 'stay in village' concept, better communication framework, and financial pooling for future exigencies. 

The measures taken by the government at large to reduce the impact of the COVID-19 pandemic was sudden, which caused unprecedented hardships leading to psychosocial stress. Our study found 'financial distress' that arose primarily out of loss of employment due to lockdown as the prime stressor. This was also evident in African countries that reported similar findings causing domestic food scarcity [9,10]. Our study in rural settings found that many families were pushed to poverty and created a psychological mindset to commit suicide. The villagers used the 'social capital' gained over the years of togetherness and a sense of belonging together with 'social networks' to overcome this adversity. This was a positive finding since the lack of it could derail the recovery process [11].

Additionally, the villagers adopted a judicious use of available resources wherein cost-cutting measures were internalized to tackle these more challenging times. A similar finding among urban residents was observed wherein APL cardholders were found suffering for want of food [6]. Apart from these positive coping mechanisms, the villagers also evinced interest to marry off their under-age daughters to get rid of daily expenses incurred on them and capitalize on the ban on large gatherings, thus incurring lesser expenditure on marriage. There was also a point mooted by the NGO working for long with these villagers. It was high time to shift from the current 'only relief' approach to the 'relief and release' approach wherein the existing schemes must generate local employment making the villagers self-reliant. This would obviate the need for villagers to venture out of their villages and thus reduce the risk of bringing COVID into the villages. He also felt that the practice of financial resource pooling envisioned by one of the studied villages could guide light to be replicated by others. 

This pandemic also brought along its share of 'surprise packages' in driving a wedge within the family bond, causing separation. This situation also enhanced the existing day-to-day problems faced by the villagers in the form of alcoholism driven gender violence and an increased domestic workload on women. No coping mechanism was adopted or suggested to tackle these issues as even the respondents trivialized these as part and parcel of their daily living.

'Fear of COVID' was reported as a common stressor among the villagers. To tackle this, they were seen to adopt measures that include an initial negative coping mechanism of 'apathy' towards the ongoing pandemic. The passage of time and influence of mass media coverage on the pandemic led to the adoption of preventive practices by all and sundry. This fear that also crept into reduced healthcare usage was tackled by adopting practices that could strengthen one's immunity. There was a unanimous opinion that 'awareness drive', especially using grass root healthcare workers whom the villagers could identify themselves with, must be done regularly in the future to minimize the chances of the villagers resorting back to callousness with regards to preventive measures.

Many of the respondents opined that the government officials of the revenue department were high-handed who were referred to as 'Kings' in their attitude. This behaviour led to a lack of confidence in the villagers in government functionaries. As a future suggestion, it was felt that there was a need to reduce the barriers in communication by bringing in attitudinal reforms of officials. It was reported that the local self-governments (gram panchayats) were kept out of the purview of COVID management and thus underutilizing their unique potentials as a key functionary at the village level. Thus, there was a felt need to bring about capacity building at the level of gram panchayats and develop strategic communication across different government functionaries to tackle the situation in a more efficient way.

Our study has a few strengths. As per our knowledge, this is the first study of its kind, which has not only tried to capture the impact of COVID in rural India but also how the villagers coped with this situation along with the suggestions for future actions in the words of people who were living through this situation. We achieved data saturation for our findings and thus achieved the required sample for the qualitative study. The study included villages situated in a single district but had varied features to COVID situational handling. The study's timeline was such that the villagers had seen various phases of lockdown, containment and phased unlock, which generated quantum of information across different genres. We have done 'member checking' to validate the data collected and have used manual content analysis, which is a gold standard for analyzing qualitative data. We also feel some of the stressors and the suggestions mooted by the key informants could be transferable across rural India. Further, we have used the COREQ checklist to report our study findings.

This study is not without limitations. There could be a loss of some information due to translation from local language to English during the preparation of transcripts, although respondent validation could have reduced it. Although focused group discussions and in-depth interviews would have added more evidence, the former could not be done due to COVID-19 safety precautions.

Although we are in complete agreement with the suggestions mooted by the village key informants, we would further like to add a few recommendations from our side: We feel that the evidence of psychosocial stress in the village community is remaining unaddressed, and despite a part of it is taken care of by the 'social capital', there is a need for employing a psychological counsellor (one per cluster of villages as feasible) who could allay the fears and psychosocial stress in a more professional way; The current rate of daily wages and the quantum of workdays under MGNREGA could be enhanced substantially to alleviate the financial distress precipitated by this unprecedented situation; The pre-occupation of government machinery in tackling the COVID crisis has led to an unnoticed increase in social evils, such as a rise in child marriages, as observed in our study. We thus recommend that such developments should not be lost sight of and addressed promptly at levels of behavioural change among villagers and by strict policing; To capitalize on the youth rendered jobless by training and employing them as village health guardians to tackle COVID related problems.

## Conclusions

The stressors due to COVID among villagers were mostly related to household level issues like financial distress, strained family bonds, gender violence and household work stress among women. The mechanisms adopted to cope with the stressors included positive mechanisms like capitalizing on social bonds, government support and exercising an economy of available resources, and harmful mechanisms like child marriages and apathetic attitudes. The suggestions for future actions included gram panchayats, adoption of the 'stay in village' concept, developing a good communication framework and financial resource pooling for future exigencies.

## Appendices

Interview Guide

Thank you for accepting our request to participate in this study. If any time during the interview you feel not to go ahead or skip any particular question or do not want to record the audio you are free to do so.

1. How the social cohesion was impacted due to fear of transmission of COVID-19? (Probes: trust issues, behaviour change).

2. Was there any loss of primary caregivers (due to death, abandonment, or lack of measures put in place if their caregivers need medical care) and if so, how the situation was dealt with?

3. How the villagers feel (Probe: dissatisfaction/ tensions) due to coercive outbreak response (viz. lockdown, quarantine, social distancing etc.)

4. Did the women face additional burdens during this period? (Probe: social duties)

5. How did you face the problems of population displacement, human rights violations, and the cancellation of events?

6. What were the causes of financial stressors?  (Probe: household expenses, restrictions, government aid)

7. How did the villagers deal with the regular health issues (Probe: available resources and personnel from routine healthcare).

8. Any problems being faced by you with the existing health systems? (Probe: Due to non-availability of healthcare workers and their ability to provide care e.g. due to fear-driven absenteeism)

9. Do you think that there has been an increase in mental health problems? ( Probe: anxiety, depression, homicide, suicide, increased alcohol abuse etc.)

10. As there is a lack of clear scientific or medical knowledge and effective treatment options, how people are dealing with this fact?

11. In your opinion how the state institutions (e.g. PRI, Police, revenue dept. or any other) could have played a better/ effective role during this period?

12. Increased sexual and gender-based violence (to be asked at the end)

Thank you for the time you spent with us and your kind cooperation in our study.
